# Evaluation of the adhesion of human dental pulp stem cells to different endodontic biomaterials before and after setting

**DOI:** 10.34172/joddd.2020.022

**Published:** 2020-06-17

**Authors:** Marzie Aghazade, Mohammad Samiei, Marjan Imani, Zahra Aghazadeh, Effat Alizadeh, Fereshte Rezaie

**Affiliations:** ^1^Department of Oral Medicine, Faculty of Dentistry, Tabriz University of Medical Sciences, Tabriz, Iran; ^2^Department of Endodontics, Faculty of Dentistry, Tabriz University of Medical Sciences, Tabriz, Iran; ^3^General Dental Practitioner, Khorram Abad, Iran; ^4^Department of Medical Biotechnology, Faculty of Advanced Medical Sciences, University of Medical Sciences, Tabriz, Iran; ^5^General Dental Practitioner, Tabriz, Iran

**Keywords:** Adult stem cells, Biocompatible Materials, MTA, Regenerative Endodontics

## Abstract

**Background.** Stem cell-based treatment modalities have been potential strategies for tissue regeneration in many conditions. Several studies have evaluated the biologic properties of DPSCs and their efficacy in the treatment of a variety of diseases. The present study was undertaken to evaluate the adhesion behavior of DPSCs on different endodontic materials before and after setting.

**Methods.** The crowns of the selected teeth were removed, and the root canals were prepared and obturated with gutta-percha and AH26 sealer. A retrograde cavity was prepared at root ends. Different materials were placed in the cavities. Then the samples were attached to the wells with the use of a chemical glue. Dental pulp stem cells were allowed to proliferate to reach a count of 2 million and transferred to -12well plates in association with a culture medium. Finally, the samples attached to the wells were exposed to the stem cells immersed in the culture medium before and after setting. Then adhesion of the stem cells was evaluated using SEM.

**Results.** The SEM results showed cellular adhesion in the samples containing CEM cement both before and after setting. The samples containing MTA Angelus and ProRoot MTA exhibited cellular adhesion before setting, with no cellular adhesion after setting. The samples containing AH26 and MTA Fillapex sealers exhibited cellular adhesion after setting, with no adhesion before setting. The samples containing simvastatin exhibited no cellular adhesion before setting; this material had dissolved in the culture medium after setting evaluation.

**Conclusion.** The results of the present study showed that of all the materials tested, CEM cement had the highest capacity for dental pulp stem cell adhesion.

## Introduction

In recent years, medical science has been revolutionized with the advent of treatment with stem cells and the production of different tissues and organs.^1-3^ Stem cells are primitive cells that can differentiate into cells with specialized functions under the influence of some specific physiologic or laboratory conditions. These cells have an unlimited capacity for proliferation and remain undifferentiated, with a capacity to differentiate into different cells in the human body.^4,5^

Stem cells from the pulp of human deciduous teeth are one of the resources for pluripotential cells;^6^ periodontal ligament is another source of cells which proliferate rapidly and express different markers of mesenchymal cells, such as CD146 and STRO-1.^7^

Stem cells isolated from the tooth pulp (DPSC) have a high potential for the treatment of disease like the bone marrow cells. These cells have the potential to differentiate into different tissues, including adipose, condylar, neuronal, bone, and smooth muscle tissues. Successful application of these cells in corneal reconstruction, capillary formation, differentiation to islet-like aggregates, and bone regeneration confirms the capacity of these cells in regenerative medicine. ^8,9^

The use of biologic factors is the prominent characteristic of clinical endodontic regenerative processes compared to conventional root canal therapy procedures.^10^ These biologic elements form blood-based scaffolds, promote endogenous growth factors in the localized environment, and induce the penetration of stem cells into the root canal system. In this context, viability, adhesion, proliferation, and differentiation of the pulp cells are necessary for the development and new pulp- and dentin-like structures. Also, these factors made the outcomes of regenerative endodontics more optimistic. Several recent studies have evaluated decreases in the concentrations of different disinfecting agents that are used during the regenerative processes. The aim is to create a stem cell-friendly environment that eliminates infection without any detrimental effects on the biologic properties of stem cells and the availability of the endogenous proteins of dentin.^3,10,11^ The dental materials used for the healing process of the root might lead to unexpected effects when they contact the periapical tissue. The filling materials or their by-products might come into contact with the surrounding tissues through the dentinal tubules and apex.

Therefore, a root canal obturating material should be biodegradable and have antimicrobial properties; it should also provide an effective seal and be stable from a dimensional viewpoint. Finally, it should have the capacity to induce cellular biologic reactions that are involved in regeneration. New studies based on cell–material reactions have revealed that dental materials should have the potential to induce regeneration or repair by inducing the stem cells of the PDL in the apical area. Cell-based studies have shown that the regeneration of pulp-like tissues and dentin occurs when they are transferred to immunosuppressed mice. Therefore, since the stem cells of the human tooth bud are derived from both the periodontal and dental pulp tissues, it would be helpful to determine cell–material reactions with the use of these cells in order to define conditions that result in the more clinical use of these materials.

For many years, different materials based on Glass Ionomer, epoxy resin, calcium hydroxide, silicone, polyketone, ZOE, and RMGI have been used to obturate the root canals. Currently, new types of obturating materials containing MTA and calcium silicate have been developed.^12,13^ Limited data are available on the effects of perfect endodontic regenerative protocols, including the use of intracanal medicaments, followed by copious irrigation with EDTA, on the attachment and proliferation of DPSC.^10^

MTA is one of the most commonly used biomaterials in regenerative endodontic treatments due to its properties, including hydrophilicity, setting in the presence of moisture, good radiopacity in vital pulp therapy, regeneration, and treatment of root perforations, in root-end filling procedures, and apexification. ProRoot MTA is a powder with very fine particles, is hydrophilic in nature, and sets in the presence of water. The hydration of the powder results in the formation of a colloidal gel which sets and creates an impermeable barrier.^13,14^

MTA Fillapex is a new calcium carbonate-based sealer with an excellent affinity for MTA-based materials due to MTA’s superb biocompatibility, bioactivity, and osteoconductivity.^15^ In 2001, MTA Angelus was introduced as a modified form of ProRoot MTA and was used in specific areas. One of the significant differences between ProRoot MTA and MTA Angelus is the absence of dehydrated calcium sulfate, one of its most important ingredients, so that MTA Angelus has a shorter setting time compared to ProRoot MTA. In addition, MTA Angelus exhibits less radiopacity compared to ProRoot MTA due to its lower content of bismuth oxide.^16^

Simvastatin improves differentiation of human pulp cells through an increase in mineralization nodules and odontogenic markers and also through angiogenic markers.^17^ On the other hand, it inhibits the proliferation of human dental pulp cells at a concentration of 1 µmol/L. This material can promote human dental pulp cell induction in the regeneration of dentin and pulp after pulpotomy procedures.^18^ AH26 is an effective sealer and is commonly used but is toxic in the root canal system. Its toxicity is attributed to the release of formaldehyde as a result of its chemical setting reactions.^19,20^

CEM cement is almost a new endodontic material introduced to dentistry by Asgary et al in 2006 for application in various root canal therapies. It is similar to MTA in practice and clinic, but it has better physical properties. After mixing with a water-based solution, it forms bioactive calcium and a phosphate-enriched mixture and then releases calcium and phosphate ions and produces a rich pool of hydroxyl ions (OH^−^), calcium ions (Ca^2+^) and phosphate ions (PO_4_^−^). These elements are used in the process of hydroxyapatite (HA) production.^21^

The present study aimed to evaluate the adhesion of human dental pulp stem cells to various biomaterials used in endodontic procedures before and after setting.

## Methods

Twenty-four human intact single-rooted teeth with an approximate root length of 10−12 mm were collected and immersed in a solution of normal saline and sodium hypochlorite for one week for disinfection. The tooth crowns were removed at CEJ with the use of a diamond fissure bur at low speed under continuous irrigation. Then the root canals were prepared and obturated with gutta-percha (Ariadent, Tehran, Iran) and AH26 sealer (Dentsply, DeTrey, Konstanz, Germany) using lateral compaction technique. Then the apical 3 mm of each root was cut away with the use of an 0.08 diamond fissure bur, and a cavity measuring 3 mm in depth was prepared at the end of the root by an ultrasonic Retrotip device. The specimens were randomly divided into six groups (n=4) in terms of the experimental materials. The samples were stored in a humid closed container until the in vitro procedures were carried out.

After retrieving the samples from the container, they were placed under UV light for 20 minutes and prepared for the placement of different endodontic materials, which consisted of MTA Fillapex (Angelus, Londrina, PR, Brazil), ProRoot MTA (Tooth-colored Formula, Dentsply, Tulsa, OK, USA), MTA Angelus (Londrina, PR, Brazil), CEM cement (Bionique Dent, Tehran, Iran), simvastatin powder (Shahr Darou, Iran) mixed with distilled water and AH26 sealer. The materials were mixed according to the manufacturers’ instructions and placed within the cavities prepared at root ends. Each endodontic material was placed in four samples. In each group, two samples were placed in contact with the solution containing stem cells at the time of mixing, and two other samples were placed in contact with the solution 24 hours after the setting of the material. All the preparation and mixing procedures of endodontic materials were carried out under a UV hood.

Two million human dental pulp stem cells were cultured in 75 flasks. DMEM, FBS, penicillin, and streptomycin comprised the culture medium of these cells. FBS and antibiotics were added to the culture medium at 10% and 1% concentrations, respectively. After the preparation of the samples, 10^5^ cells were added to each well of the culture medium. The prepared roots were attached with a chemical glue to the wells twice (immediately after mixing = unset and 24 hours after mixing = set) and immersed in the medium in the wells. Scanning electron microscope (SEM) images (MIRA3 FEG-SEM, Czech Republic) were prepared one week after placing the roots in contact with the cells. To this end, after drying the samples in a clean chamber at 25−35ºC, they were coded and mounted on the stent with a carbon or copper label, followed by placing within a gold-coating machine for 300 seconds to cover the surface of the root end with a thin layer of gold. Then, SEM images were taken under a scanning electron microscope at different magnifications.

## Results

**Figure 1:**
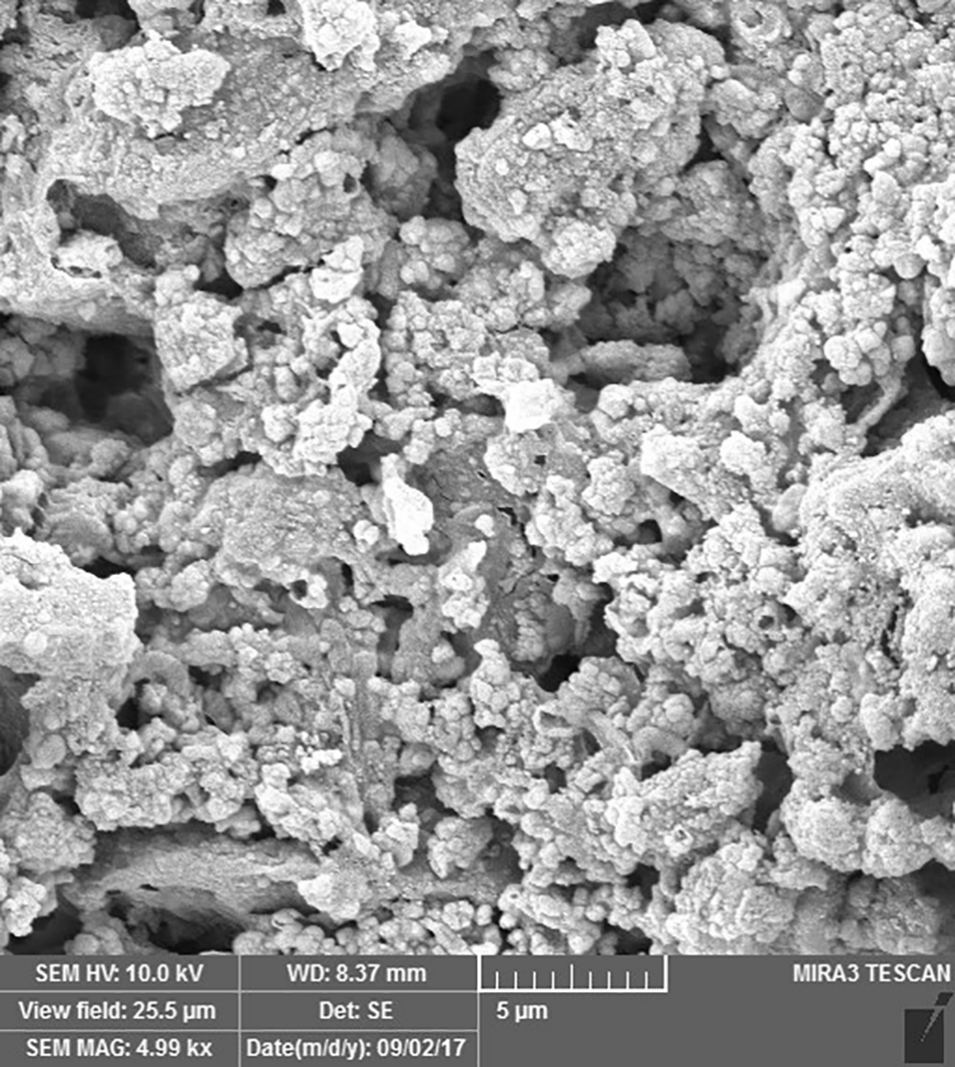
Cellular adhesion in the unset CEM cement sample.

**Figure 2:**
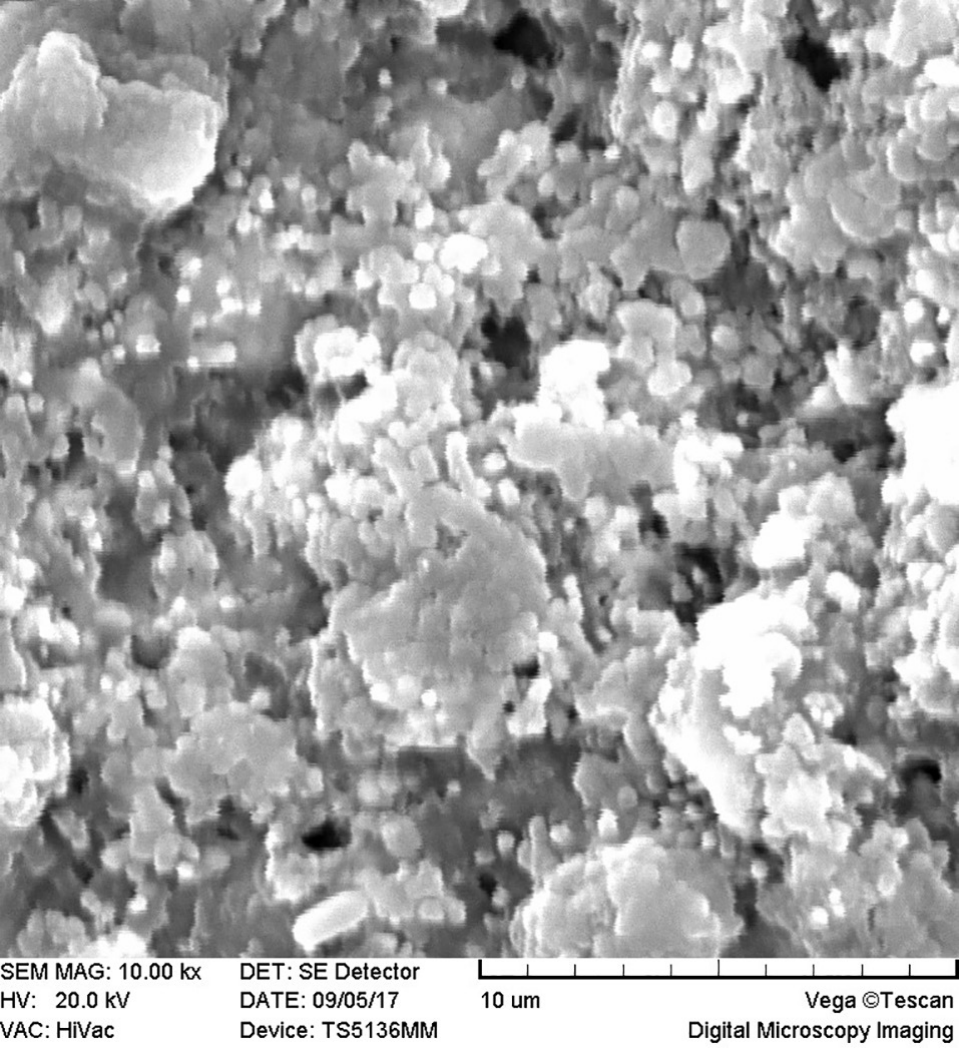
Cellular adhesion in the set CEM cement sample.

**Figure 3:**
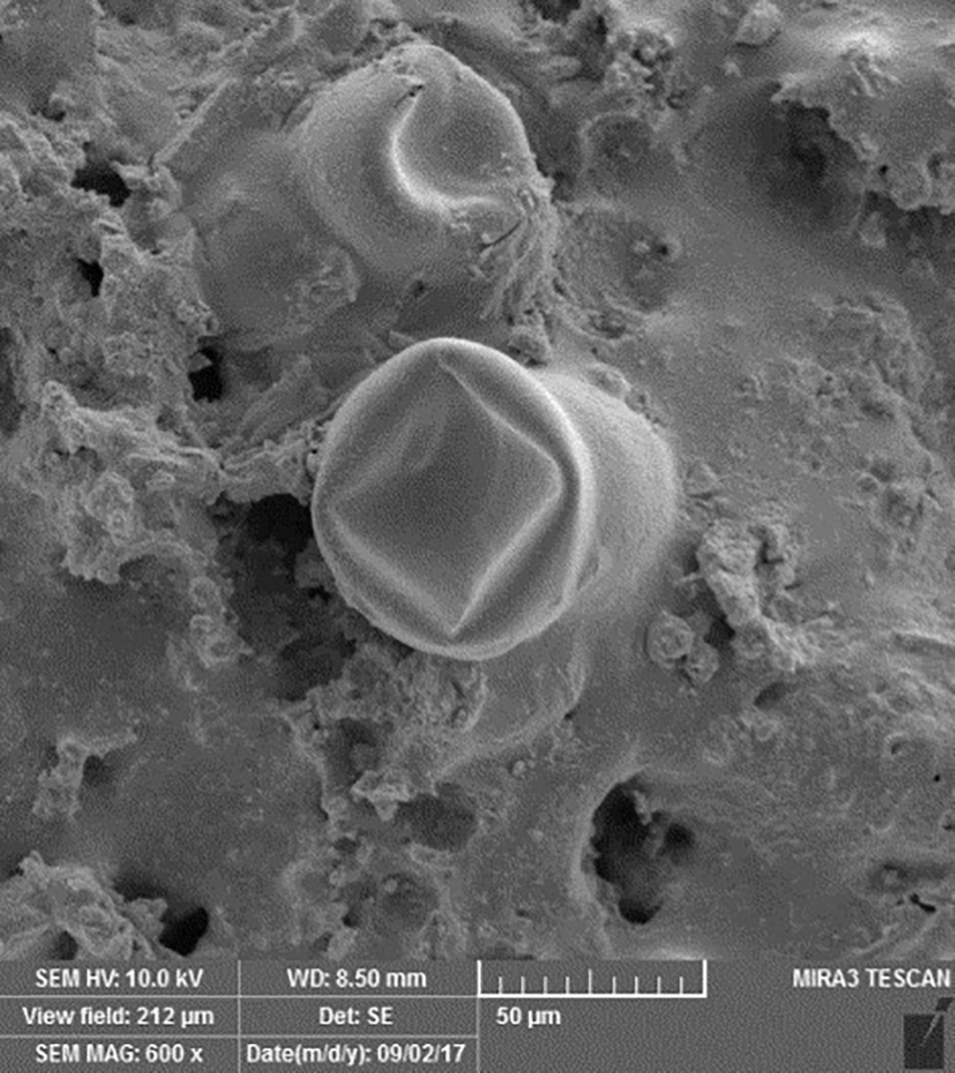
No cellular adhesion in the unset AH26 sample.

**Figure 4:**
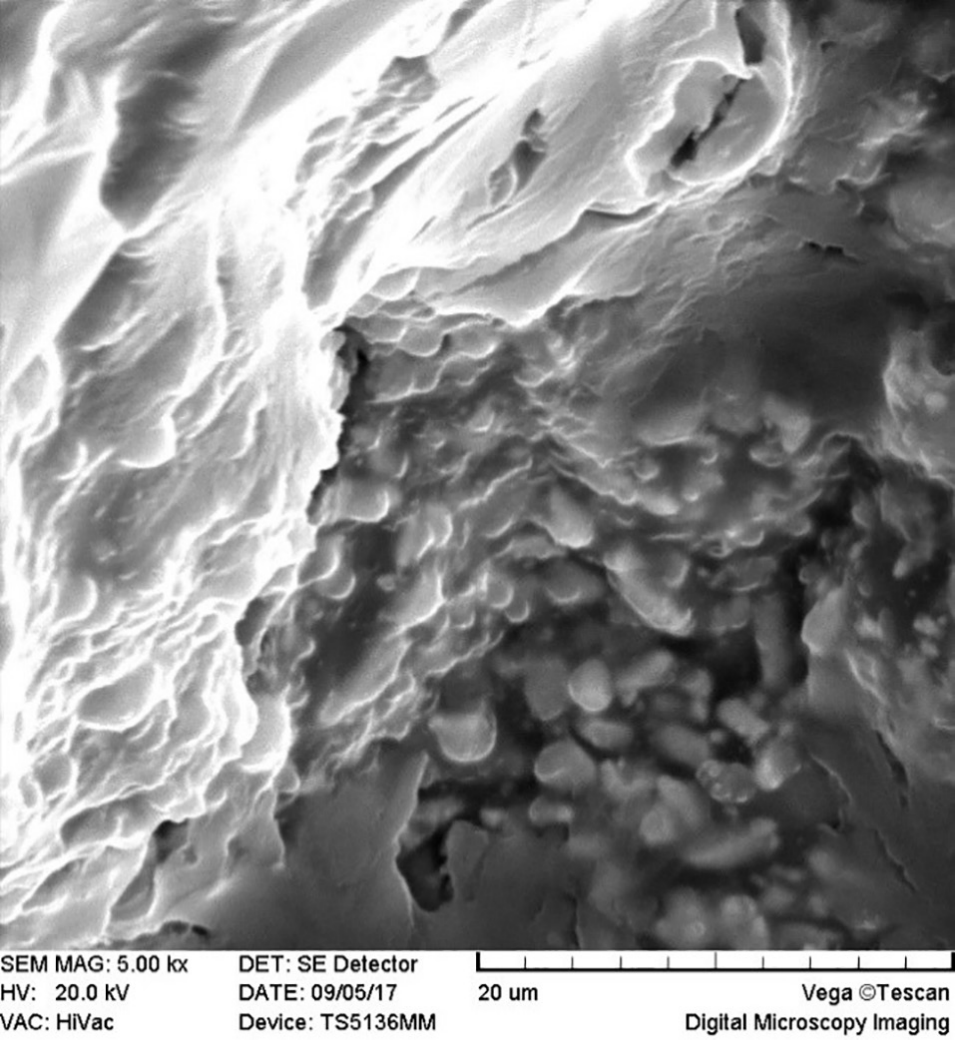
Cellular adhesion in the set AH26 sample.

**Figure 5:**
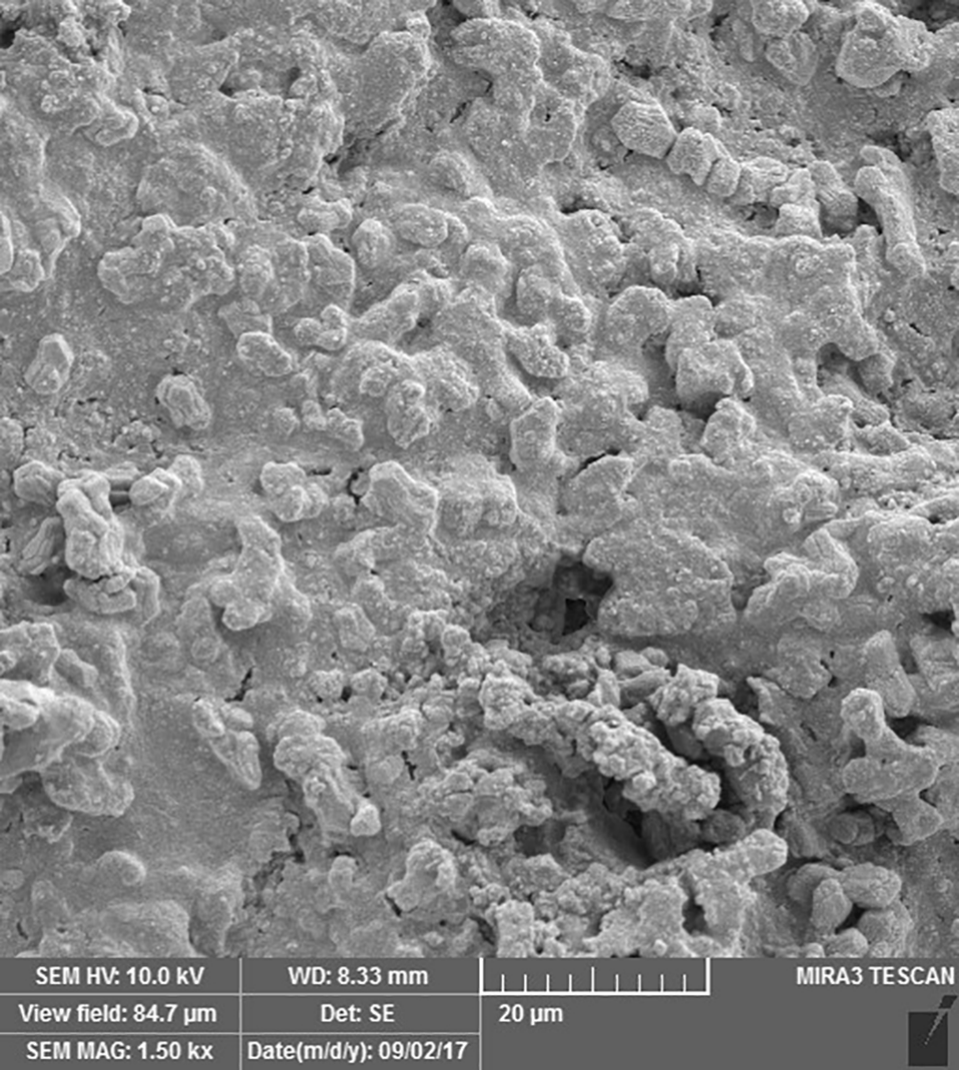
No cellular adhesion in the unset MTA Fillapex sample.

**Figure 6:**
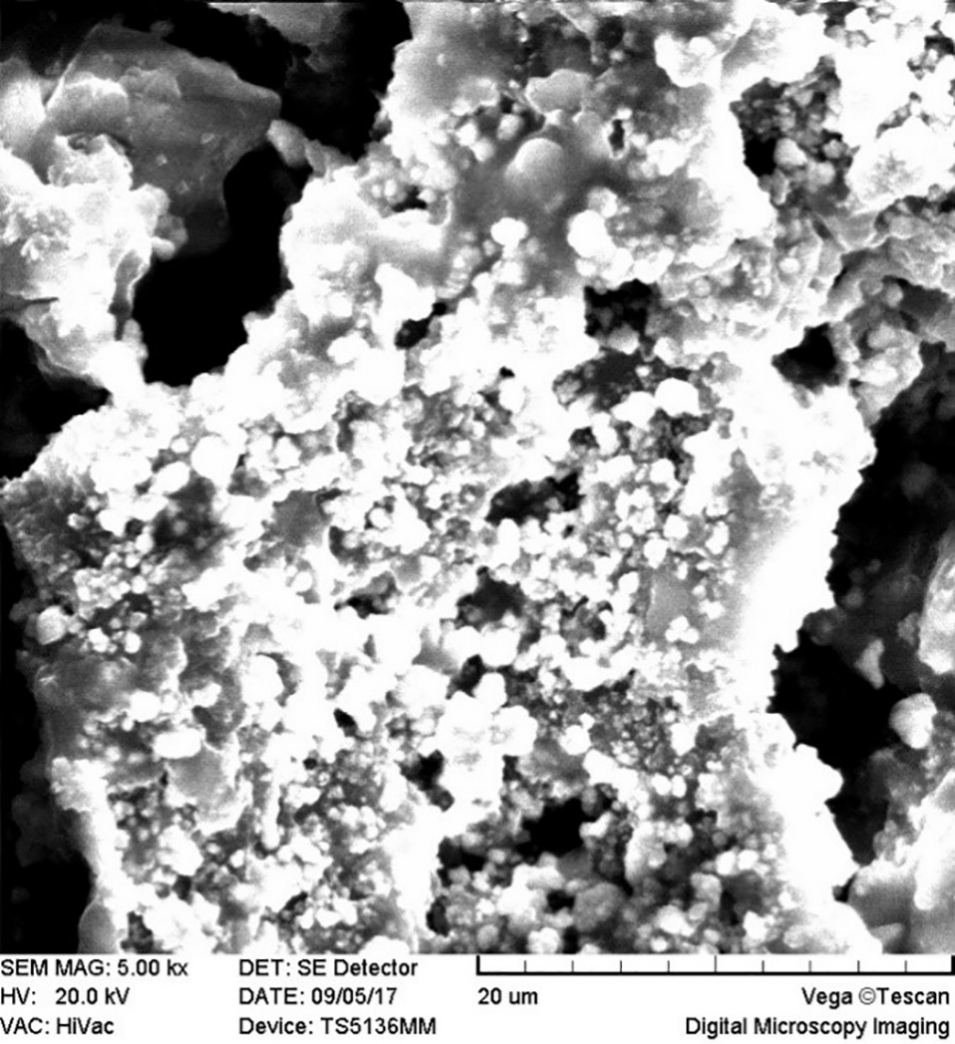
Cellular adhesion in the set MTA Fillapex sample.

**Figure 7:**
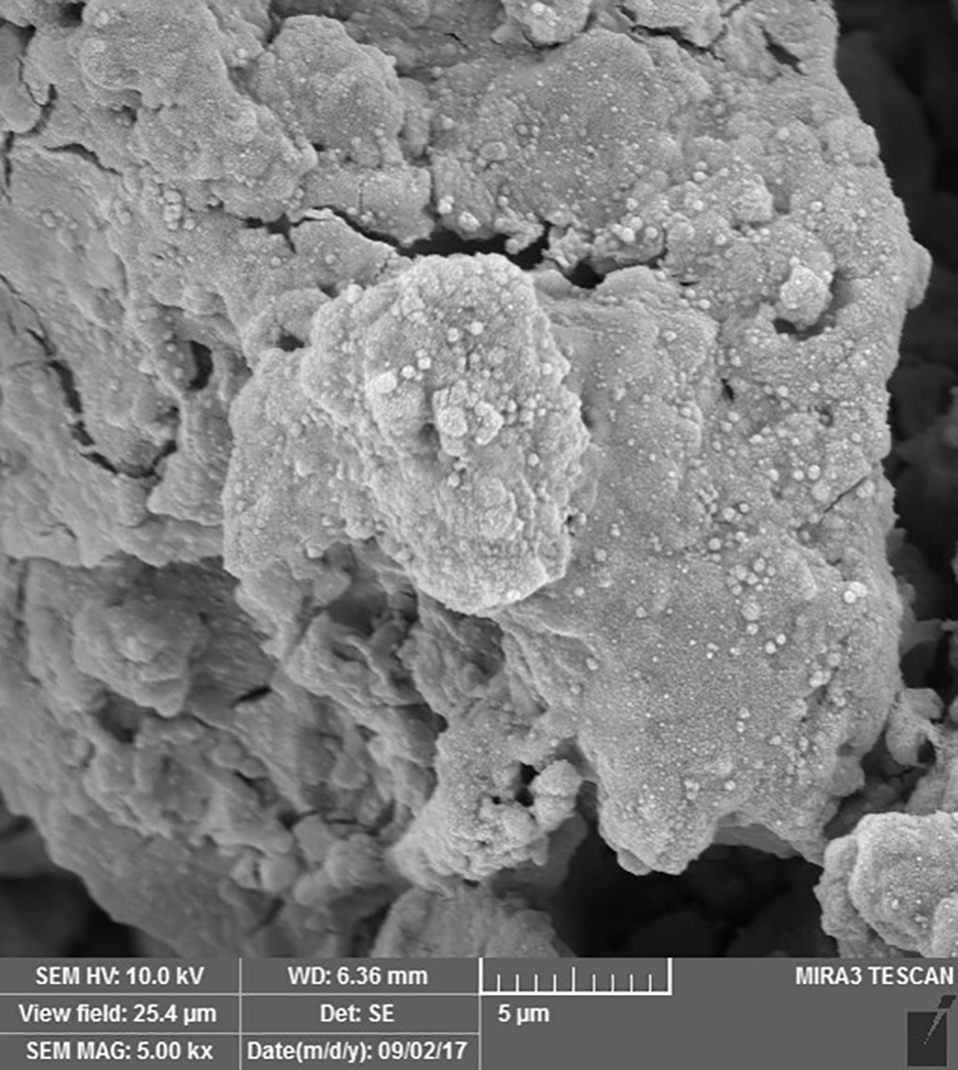
Cellular adhesion in the unset MTA Angelus sample.

**Figure 8:**
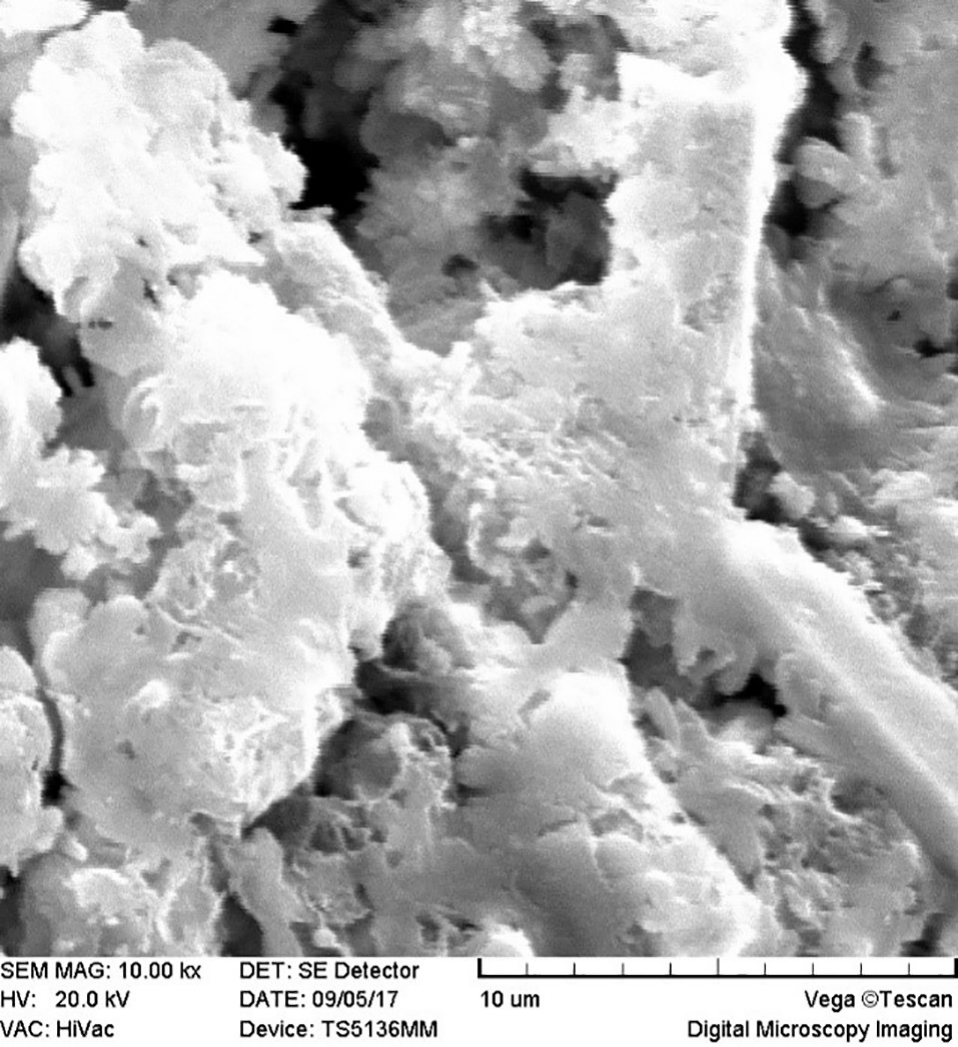
No cellular adhesion in the set MTA Angelus sample.

**Figure 9:**
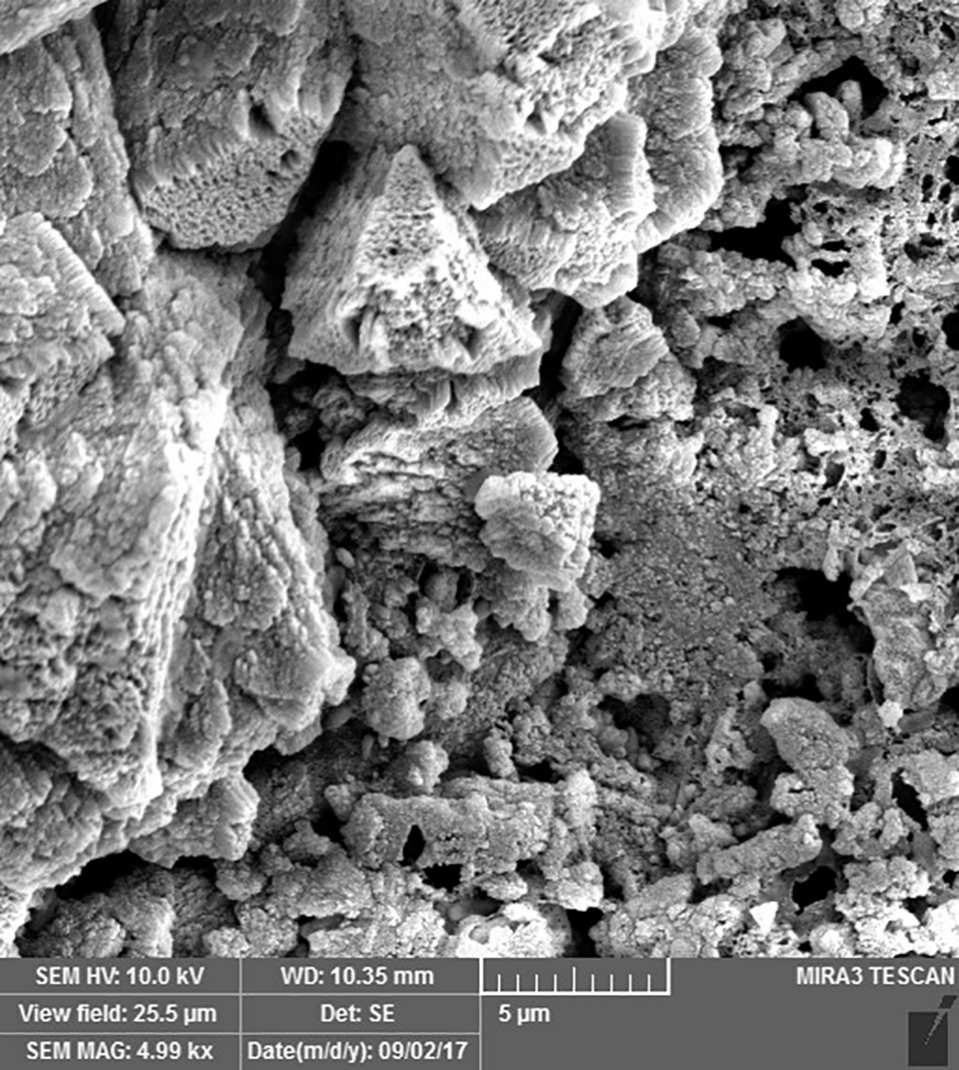
Cellular adhesion in the unset ProRoot MTA sample.

**Figure 10:**
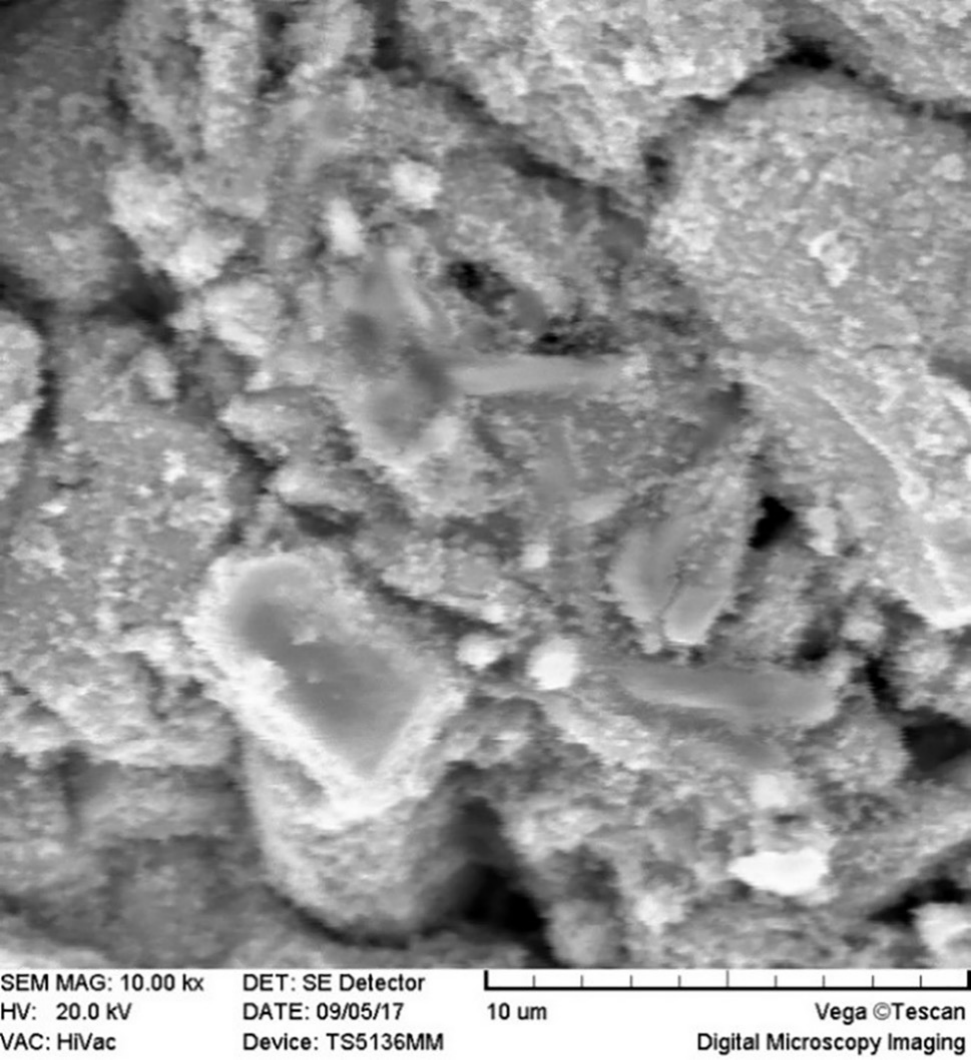
No cellular adhesion in the set ProRoot MTA sample.

SEM images revealed cellular adhesion in CEM cement-containing samples in both the set and unset conditions (Figures 1 and 2). In samples containing AH26, no cellular adhesion was detected in the unset samples (Figures 3 and 4); however, in the set samples, cellular adhesion was shown, which was similar to the MTA Fillapex-containing samples (Figures 5 and 6). Concerning MTA Angelus and ProRoot MTA, cellular adhesion was observed in samples that were placed in contact with the stem cell-containing solution before setting; however, no adhesion was observed in any of the samples after setting (Figures 7-10). Concerning simvastatin, no cellular adhesion was observed in any sample before setting. Also, this material had dissolved in the solution and washed away from the retrograde cavity in the evaluation carried out after setting.

## Discussion

In the present study, 12-well plates with very low adhesion were used to increase the adhesion of dental pulp stem cells to the endodontic materials compared to the well walls. These containers were covered with a layer of hydrogen with a neutral charge to keep the cells in a suspended and unattached status; these containers have been used in several studies on stem cells.^10^ In the present study, the stem cells were placed in direct contact with endodontic materials with no need for a scaffold or dentin. In this context, the stem cells were immersed in a culture medium containing FBS, DMEM, penicillin, and streptomycin, and the samples which had received endodontic materials at their root ends were immersed, too, which is consistent with the clinical scenario in regenerative endodontics. A hypothesis that can explain a lack of adhesion and dissolution of simvastatin, in contrast to its non-toxic nature, is that the suspension might have caused physicochemical changes on the surface of endodontic materials.

Recent studies have shown that the viscoelastic properties of a precursor change the ability of the cells to attach, be distributed, and differentiate significantly.^10^ Furthermore, differences in laboratory conditions might explain these differences in the adhesion of stem cells to different endodontic materials in studies. Another hypothesis that can explain the differences in cellular adhesion between these materials is that some of these materials might pave the way for greater adhesion of these cells by creating surface roughness. In the present study, UV radiation was used to disinfect the samples, which might not have a negative effect on the surface properties of dentin and endodontic materials, in contrast to autoclaving. Lymperi et al^22^ reported that tooth pulp stem cells grew on the surface of MTA, dentin chips, and Bio-Oss, with the minimum cells growing on Bio-Oss, indicating a much higher ability of MTA and dentin chips to attract tooth pulp stem cells; therefore, they are better candidates for bioengineered pulp regeneration. In the present study, MTA Angelus and ProRoot MTA exhibited cellular adhesion when they contacted stem cells, consistent with the study above. Agrafioti et al^23^ evaluated reactions between dental pulp stem cells and MTA and Biodentine after their contact with different environments and concluded that the viability of cells 24 hours after MTA setting was significantly higher compared to that one hour after setting of MTA. Placing endodontic materials in an acidic environment, such as citric acid, increased the rate of adhesion and survival.In addition, in the present study, MTA Angelus and ProRoot MTA exhibited cellular adhesion when they contacted the stem cells. In a study by Guven et al^20^ on the interaction between tooth bud stem cells and calcium silicate-based cements, it was shown that one day after the experiment, only MTA Fillapex exhibited cytotoxicity compared to the negative control group. The SEM analysis showed the highest surface adhesion in the iRoot SP and the control groups. After 24 hours, the number of cells adhering to the surface decreased in the MTA Fillapex group, which is different from the results of the present study, in which there was cellular adhesion in the set samples. In another study, the effects of simvastatin in association with α-TCP, in comparison with MTA, were evaluated on the odontoblastic differentiation of tooth pulp stem cells, and it was shown that the cellular growth and ALP activity on the simvastatin + αTCP group was higher than that in the MTA group.^24^ No adhesion was detected in any of the groups before and after setting. In a study by Catala et al^25^ the viability of HDPSCs after 24−48 hours on MTA Repair and NeoMTA Plus was at a moderate level; however, at 48 and 72 hours, Biodentine exhibited higher viability compared to the two materials above. Moreover, studies have shown a higher rate of cell proliferation and adhesion on Biodentine disks, with moderate rates on MTA Repair and NeoMTA Plus disks.

In a study by Ahmed et al,^26^ white MTA (WMTA) and CaCl_2_ were compared. WMTA exhibited favorable cellular adhesion and cytotoxicity, while fast-setting WMTA exhibited moderate to severe toxicity at three consecutive concentrations, and the cellular adhesion properties were less favorable. However, DPSCs treated with fast-setting WMTA extracts exhibited superior expressions of dentinogenic gene markers than WMTA. Furthermore, DSPP was upregulated only in WMTA on day three compared to days one and seven in fast-setting WMTA. Consequently, the addition of CaCl_2_.2H_2_O enhances the cytotoxicity but improves the dentinogenic differentiation potential of WMTA on DPSCs.

A study by Mohamed et al^27^ on the comparison of proliferation and viability of HDPSCs on MTA, CEM cement, and Nano-hydroxyapatite (NHA) revealed a lower rate of proliferation and a higher rate of toxicity on NHA compared to the two other materials.

## Conclusion

The results of the present study showed that samples with CEM cement at their root ends in both the set and unset conditions exhibited cellular adhesion. Generally, CEM cement exhibited a higher capacity for adhesion of stem cells compared to the other biomaterials.

## Authors’ contributions

The concept and the design of the study were developed by MS and FR. The isolation and culture of human dental pulp stem cells were carried out by MI and MA. Data entry and statistical analyses were carried out by EA, MA, and ZA. The manuscript was written by MA and FR. The proofreading was carried out by MS and FR. All the authors participated in the literature review.

## Acknowledgments

The authors especially thank the staff of the Stem Cell Research Center, Tabriz University of Medical Sciences, for their assistance in carrying out the study.

## Funding

This study was written based on a dataset of a DDS thesis, registered and financially supported by the Stem Cell Research Center, Tabriz University of Medical Sciences, Tabriz, Iran.

## Competing interests

The authors declare no conflict(s) of interest related to the publication of this work.

## Ethics approval

The Ethics Committee of Tabriz University of Medical Sciences (TUOMS) approved the protocol of this study, which was in compliance with the Helsinki Declaration (Ethics code: IR.TBZMED.REC.1395.1037).
